# Increasing Awareness about Antibiotic Use and Resistance: A Hands-On Project for High School Students

**DOI:** 10.1371/journal.pone.0044699

**Published:** 2012-09-12

**Authors:** Maria João Fonseca, Catarina L. Santos, Patrício Costa, Leonor Lencastre, Fernando Tavares

**Affiliations:** 1 Faculdade de Ciências, Departamento de Biologia, Universidade do Porto, Porto, Portugal; 2 IBMC - Instituto de Biologia Molecular e Celular, Universidade do Porto, Porto, Portugal; 3 CIBIO - Centro de Investigação em Biodiversidade e Recursos Genéticos, Universidade do Porto, Vairão, Portugal; 4 Faculdade de Psicologia e de Ciências da Educação, Universidade do Porto, Porto, Portugal; Los Angeles Biomedical Research Institute, United States of America

## Abstract

**Background:**

Health-promoting education is essential to foster an informed society able to make decisions about socio-scientific issues based on scientifically sustained criteria. Antibiotic resistance is currently a major public health issue. Considering that irrational antibiotic use has been associated with the development and widespread of antibiotic resistant bacteria, educational interventions to promote prudent antibiotic consumption are required.

**Methodology/Principal Findings:**

This study focuses on the outcomes of an interventional program implemented at the University of Porto, Portugal, to promote awareness about antibiotic resistance at high school levels (15–17 year old). The project *Microbiology recipes: antibiotics à la carte* articulates a set of wet and dry lab activities designed to promote the participants’ understanding of concepts and processes underlying antibiotics’ production and activity, such as the notion of mechanisms of action of antibiotics. Following a mix-method approach based on a pre−/post design, the effectiveness of this project was assessed by gathering data from surveys, direct observation and analysis of artifacts of 42 high school students (aged 15 and 16 years). The results indicate that the participants developed a more comprehensive picture of antibiotic resistance. The project was shown to promote more sophisticated conceptualizations of bacteria and antibiotics, increased awareness about the perils of antibiotic resistance, and enhanced consciousness towards measures that can be undertaken to mitigate the problem. The participants regarded their experiences as enjoyable and useful, and believed that the project contributed to improve their understanding and raise their interest about the issues discussed. Furthermore, there were also improvements in their procedural skills concerning the laboratory techniques performed.

**Conclusions/Significance:**

This study evidences the possibility of increasing high school students’ awareness about the consequences of antibiotic resistance and the importance of judicious antibiotic use. The findings inform about the educational benefits of incorporating hands-on activities in science education programs.

## Introduction

The development of antibiotic drugs provided the basis for infectious disease control. However, with the emergence and widespread of drug-resistant and multidrug resistant bacteria, antibiotic resistance has become a major public health issue. Paradoxically, the overuse and misuse of antibiotic drugs have scaffolded this turn of events in the warfare against bacterial infectious disease [1], [2]. Unreasonable antibiotic consumption in human healthcare and farm animal husbandry increases the selective pressure for resistant bacteria, accelerating the rhythm at which resistance spreads. In this context, as emphasized by the World Health Organization, in the report “Overcoming antimicrobial resistance” [3] and 11 years later by selecting the theme “Combat Drug Resistance” for World Health Day 2011, it is necessary to promote informed decision-making about antibiotic consumption. Accordingly, the calls for public health education programs have resulted in numerous educational resources, such as the ones made available by the Centers for Disease Control and Prevention (http://www.cdc.gov/drugresistance/campaigns.html) and the Alliance Working for Antibiotic Resistance Education (http://www.aware.md/). Nevertheless, reliable indicators of the efficacy of most of these resources are still missing [4]. Furthermore, studies show that the general public remains unaware of basic aspects related with antibiotics’ modes of action, and frequently engage in misinformed behaviors [5], [6]. This reinforces the importance of developing health education programs to promote appropriate antibiotic use and enhance public understanding about antibiotic drugs.

Considering that educational programs targeting young people expectably contribute to a future generation of scientifically literate antibiotic users, the purpose of this study was to develop, implement and assess a hands-on interventional program to promote awareness about antibiotic resistance at high school levels (ages 15–17 years). The main goal of the project *Microbiology recipes: antibiotics à la carte* was to promote the participants’ understanding of biological concepts and processes underpinning the notion of antibiotic production and activity, by eliciting their engagement in microbiology procedures.

### Microbiology Recipes: Antibiotics à La Carte

This study focuses on the outcomes of the project *Microbiology recipes: antibiotics à la carte*, implemented in the scope of Porto’s Junior University (UJr) (http://universidadejunior.up.pt/index.php/paginas/english/home). As a member of the European Children’s University Network (EUCU.NET, https://sites.google.com/site/eucunetevents/), UJr is a summer school-based initiative that seeks to promote Science & Technology), Arts, Humanities and Sports education amongst elementary and high school students (aged 11 to 17). Each year, a list of projects designed by university lecturers and implemented by undergraduate and graduate students within the university facilities is made available in UJr’s website, so that interested students can chose and apply to the one(s) they prefer.

*Microbiology Recipes: antibiotics à la carte* was developed as a one-week long inquiry-based hands-on project for high school students. Whereas traditional educational interventions to decrease antibiotic use and improve knowledge about antibiotics have mainly relied on information campaigns and state or nationwide programs [4], [7], [8], [9], [10], [11], practical activities in this scope are much scarcer [12], [13], [14], [15]. Practical work, generally understood as activities that demand an active engagement in the manipulation of objects and materials [16], [17], [18], has been known to scaffold students’ learning by: sparking their interest [17], [19]; fostering social interaction [18], [19]; and promoting scientific reasoning by allowing to make connections between observable phenomena and the underlying ideas [16], [19], [20]. Practical work can be an efficient strategy to promote students’ knowledge about antibiotics and antibiotic resistance, considering that at high school levels, the understanding about these concepts can be compromised by: their abstract nature; misconceptions about the notion of microorganism [21], [22]; and difficulties in transitioning between micro and macro levels of conceptualization [23].

To meet these concerns, the project’s instructional design was purposely built upon a practical component, following the adaptation and extension of a practical activity on the antibiotic effect of natural phytoactive compounds [24].

**Figure 1:**
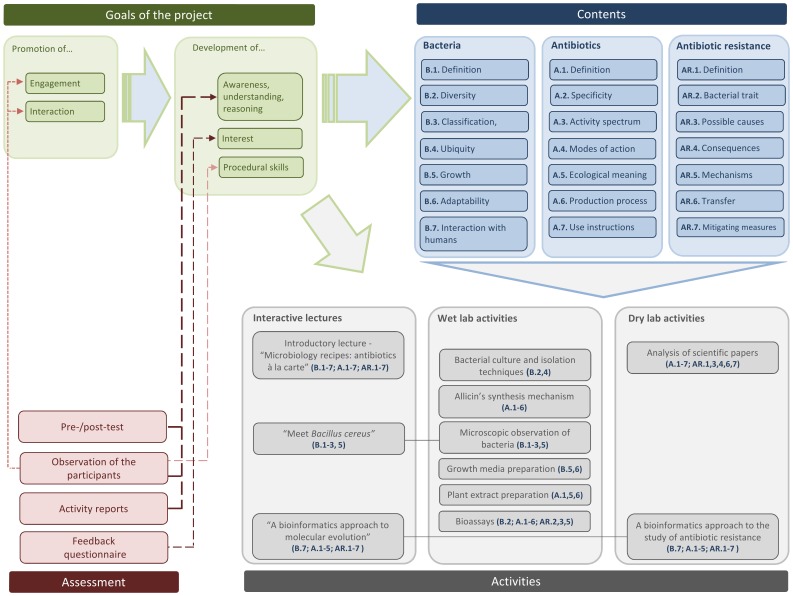
An holistic perspective of the project’s rationale and implementation. The goals of the project, the contents covered, the activities carried out, and the assessment instruments used in the study were purposely articulated to provide a comprehensive depiction of its educational effectiveness.

The project began by an introductory session in which its scope and aims, the activities to perform, and basic laboratory safety rules were presented to the participants. Following this introduction, as outlined in Fig. 1, the participants took part in integrated activities, aligned with the goals of the project and with the contents addressed: three interactive lectures, six wet lab activities, and two dry lab activities.

The contextualization of the project was made during an interactive lecture covering scientific notions and concepts related to bacteria, antibiotics and antibiotic resistance that have been discussed as pivotal in previous research studies [22], [25], [26], [27], including: bacteria’s growth, adaptability, and ubiquity; antibiotics’ activity spectrum, modes of action, and production processes; and the causes and consequences of bacterial resistance to antibiotics (Fig. 1).

Two additional lectures were used to introduce two practical activities. One of these - Meet *Bacillus cereus* provided the theoretical background for a wet lab activity on bacterial growth and diversity (Fig, 1), in which the participants made and observed slide preparations of bacterial cells at different growth stages.

The other interactive lecture introduced a bioinformatics exercise addressing the evolution of a gene coding for resistance to an antibiotic (Fig. S1-B). The purpose was to acquaint the participants with tools commonly used in research with which they were not familiar. Most importantly, it was intended to reinforce the significance of antibiotic resistance, by providing an evolutionary perspective.

The other dry lab activity involved the analysis of scientific papers, carried out with the main objective of illustrating how research findings are reported within the research community, and discussing published scientific evidence about the widespread of antibiotic resistance, antibiotic use behaviors, and natural compounds as alternatives or coadjuvants of commercial antibiotics.

In one of the remaining five wet lab activities, the participants practiced bacterial culture and isolation techniques, by collecting and growing environmental samples from various sources, including door knobs, keyboards, cellphones, foodstuffs, plant leafs, and their hands and nostrils. The main goal was to witness the diversity and ubiquity of bacteria.

In another session, the participants discussed the biosynthesis process of allicin, the major phytoactive compound to which garlic’s antibiotic properties are ascribed, while carrying out a practical activity that demonstrates that garlic’s antibiotic compounds are induced upon injury (Fig. S1-A). They were introduced to several measures required to handle such volatile, unstable substances and reflect about the ecological meaning of the production of compounds with antibiotic properties, especially amongst bacteria.

The participants were also engaged in the preparation of growth media. Besides the development of procedural skills, this was expected to foster the acknowledgement that bacteria have specific requirements that determine their growth.

Another activity involved the preparation of plant extracts from organisms described in the literature as having antibiotic properties, some of which were brought to the laboratory by the participants. The tasks performed were expected to give an idea of the complexity involved in screening and testing natural antibiotics.

Using the agar diffusion method, biossays were performed with the extracts previously prepared and with commercial antibiotics, to allow a comparative overview. The participants observed, registered and discussed the results obtained, and prepared a brief activity report, which was discussed, aiming to summarize the key points of the contents addressed and the procedures carried out throughout the project.

A comprehensive overview of the project’s rationale, including the contents addressed, is available in Fig. 1, and the schedule and distribution of the activities throughout the week is presented in Fig. S2.

To evaluate the effectiveness of *Microbiology recipes: antibiotics à la carte*, an assessment strategy combining qualitative and quantitative approaches was set up (Fig. 1). The main research question driving this investigation was: are there significant changes in high school students’ awareness and understanding about antibiotic use and resistance as the result of their participation in this hands-on project? The study also sought to determine the impact of the project on the participants’ interest about the topics addressed and their procedural competencies. Among the findings obtained, it was noticed that the project impacted positively on the participants’ awareness and understanding about bacteria, antibiotics, and antibiotic resistance. The innovative nature of the project, concerning the implementation setting and the combination of activities ranging from bioinformatics to natural antibiotic testing, contributed to dismiss misconceptions, enhance the sense of self-responsibility, and promote the development of procedural skills.

## Methods

### Participants

The study involved 42 high school science students who participated in the project in 2010 (*n* = 25) and 2011 (*n* = 17). The participants (30 females, 12 males, aged 15 and 16 years) had just finished grades 10 (*n = *28) and 11 (*n* = 14). All of them enrolled in the project by their own initiative, by registering online at UJr’s website, and following the procedures required. As most of UJr’s applicants are underage, the parents’ consent is formalized through the payment of a registration fee. Taking into account all ethical requirements, each project is institutionally approved by both UJr’s organizing committee and the board of direction of each Faculty where the activities take place. For the purpose of this study, the assessment of the project’s outcomes was depicted in the project’s planning, together with the outline of every activity proposed. Upon entering the project, the participants were invited to take part in the study and informed of its nature and aims. They were given the chance to participate in the project without participating in the study, and to withdraw from the latter should they wish to. All the data collected were processed and analyzed anonymously.

According to the Portuguese biology curricula, the 10^th^ and 11^th^ grade biology and geology programs include contents related, respectively with *cell structure* and *uni−/multicellularity* [28], *taxonomy and classification systems* [29]. However, the concept of antibiotics is only addressed at 12^th^ grade biology [30]. Therefore, in spite of being science students, these participants were not expected to be particularly knowledgeable about antibiotic resistance as a result of their formal education.

### Data Collection

To obtain a broader, more inclusive depiction of the effectiveness of the project, a mixed methods approach was used [31], combining questionnaire analysis, observations, and analysis of activity reports (Fig.1). Qualitative and quantitative data were gathered on the participants’ understanding, reasoning, interest and procedural skills. Considering the reported influence that students’ engagement and interaction with their peers has on learning [18], [19], [32], [33], these two aspects were also evaluated.

To assess the participants’ understanding and reasoning about bacteria, antibiotics and antibiotic resistance, an open-ended pre−/post-test was developed (Table 1), considering topics available in previous studies [5], [6], [26], [34], [35]. Common misconceptions such as antibiotic use for flu treatment [5], [26], [34], or the notion that resistance refers to a characteristic of the host organism [25] were considered.

**Table 1:** Pre−/post-test used to assess the participants’ understanding and beliefs about bacteria, antibiotics and antibiotic resistance.

Q1.	How do you define bacteria?
Q2.	Are bacteria beneficial or harmful for humans? Give some illustrative examples.
Q3.	Describe the main phases in bacteria’s growth cycle.
Q4.	Do you think that bacterial infectious diseases are currently under control? Justify your answer.
Q5.	How do you define antibiotics?
Q6.	How do you explain the selectivity of antibiotics for microorganisms?
Q7.	Imagine that you have the flu, you are feverish and aching. In this situation, do you think that antibiotic prescription would be a suitable solution? Justify your answer.
Q8.	Describe how an antibiotic is produced.
Q9.	How do you define antibiotic resistance?
Q10.	List measures that can be used to avoid or reduce antibiotic resistance.
Q11.	Do you agree with the statement: The progeny of antibiotic resistant bacteria is also resistant? Justify your answer.

Naturalistic observations [36] were carried out to identify misconceptions and reasoning difficulties, and to evaluate the participants’ interaction and engagement. The participants prepared activity reports, which were also examined, with the main purpose of assessing how they interpreted, explained and discussed their findings.

Finally, a self-reported questionnaire with closed and semi-open questions was used to gather the participants’ feedback about their experience in the project (Table 2). Based on previous studies on students’ views about effective practical work lessons [18], [19], particular attention was given to the interest, difficulty, usefulness and meaningfulness of the activities.

**Table 2:** Feedback questionnaire.

**Rate the following aspects on a scale from 1 (Very low/Not at all…) to 5 (Very high/Completely…)**	
• Organization and structuring of the contents	• Effort required
• Difficulty of the contents	• Contribution to understand the issues discussed
• Interest of the contents	• Contribution to critically reflect about the issues discussed
• Difficulty of the techniques	• Contribution to enhance the curiosity about the issues discussed
• Articulation between content and techniques	• Overall satisfaction about the project
• Suitability of materials used	
**List the…**	
… most positive aspects (open question)	… less positive aspects (open question)
**Make the comments and suggestions you find necessary (open question)**	
**Evaluate the project in a scale of 1 (Mediocre) to 5 (Excellent)**	

### Data Analyses

Questionnaire data were recorded, codified and categorized. The content analysis of the participants’ responses to the open questions was performed following the recommendations available in Krippendorff [37] and Weber [38].

The analyses of the participants’ pre- and post-test responses were conducted with the purpose of measuring the range of impact of the project, and unveiling the qualitative variations in the participants’ reasoning. Besides determining the number of students who provided correct and incorrect responses, the content of those responses was scrutinized. For every response, the number of correct and incorrect notions was quantified, and their pre−/post-test variation measured. To gauge changes in the participants’ reasoning, coding rubrics were developed for each question (See Table S1), informed by Bloom’s taxonomy of cognitive domains, a classification system that categorizes cognitive thinking skills according to levels of abstraction [39], [40], [41]. The interpretation of the participants’ responses was based on previously defined guidelines [41], [42].

Using IBM SPSS Statistics 20, descriptive and inferential statistical analyses were performed to examine and compare the responses obtained. One sample *t*-tests were used to examine the mean scores for the items measured on five point Likert-type scales. Scores below, equal or above the midpoint of the scale (test value = 3), were respectively considered indicative of negative, neutral or positive responses, for a 95% confidence interval. For the open-ended questions, paired samples *t*-tests were used to compare the pre−/post-test variation in the number of correct/incorrect notions provided per response, and in the rubric scores. Variations were considered significant for *p*<0.05. The strength of the mean differences registered was measured using Cohen’s *d* [43]. Effect sizes equal to 0.2, ranging from 0.5 to 0.8, or above 0.8, were respectively considered small, medium or large [43], [44]. For the responses codified as dichotomous variables (e.g. a “Don’t know” answer), the McNemar test was used to compare pre-test and post-test scores [45].

## Results

### Pre- and Post-test Performance

The data collected point towards the improvement of the participants’ understanding of the concepts of bacteria, antibiotics and antibiotic resistance, and of their awareness about bacterial infectious disease control, antibiotic use and bacterial resistance to antibiotics.

Significant pre−/post-test differences were observed for every question in the questionnaire (*p*<0.05). There were significant improvements in the quality of the participants’ responses, as demonstrated by the enhancement in the rubric scores for the eleven questions presented (Table S2; see Table S1 for details on the pre−/post-test scoring rubrics). For most questions, there was an increase in the number of students able to achieve top-level responses in the post-test (Q1: 1 *vs.* 11; Q3: 0 *vs.* 13; Q4: 0 *vs*. 5; Q5: 0 *vs.* 6; Q7: 1 *vs.* 16; Q9: 12 *vs.* 23; Q10: 0 *vs.* 3). This improvement can be ascribed to:

the increase in the amount of correct notions or valid claims provided per response for every question presented (See Table S2);
the decrease in the amount of incorrect notions or invalid claims provided per response for questions Q1, Q4, Q5, Q7, and Q9 (See Table S2).

There was also an increase in the number of participants conveying correct notions in questions Q1, Q2, Q3, Q5, Q6, Q8, Q9, Q10 and Q11 (See Table S3), and a decrease in the number of participants who did not answer questions Q3 (27 *vs.* 0, *χ* ^2^(1) = 23.04, *p*<0.001), Q6 (20 *vs.* 5, *χ* ^2^(1) = 11.53, *p*<0.001), Q8 (33 *vs.* 9, *χ* ^2^(1) = 22.04, *p*<0.001), Q10 (11 *vs.* 0, *χ* ^2^(1) = 9.09, *p*<0.001), and Q11 (19 *vs.* 3, *χ* ^2^(1) = 14.06, *p*<0.001).

### Observation of the Participants

#### Misconceptions and difficulties

No relevant difficulties or misconceptions were identified during the activities, although several participants admitted that they “did not know the human body harbors so many bacteria” and that antibiotic drugs affect bacteria from the human microbiota. Also, whilst most of them knew that edible plants and herbs may produce substances with pharmacological interest, they did not know that plant extracts can be used to inhibit bacteria.

#### Procedural competencies

**Figure 2:**
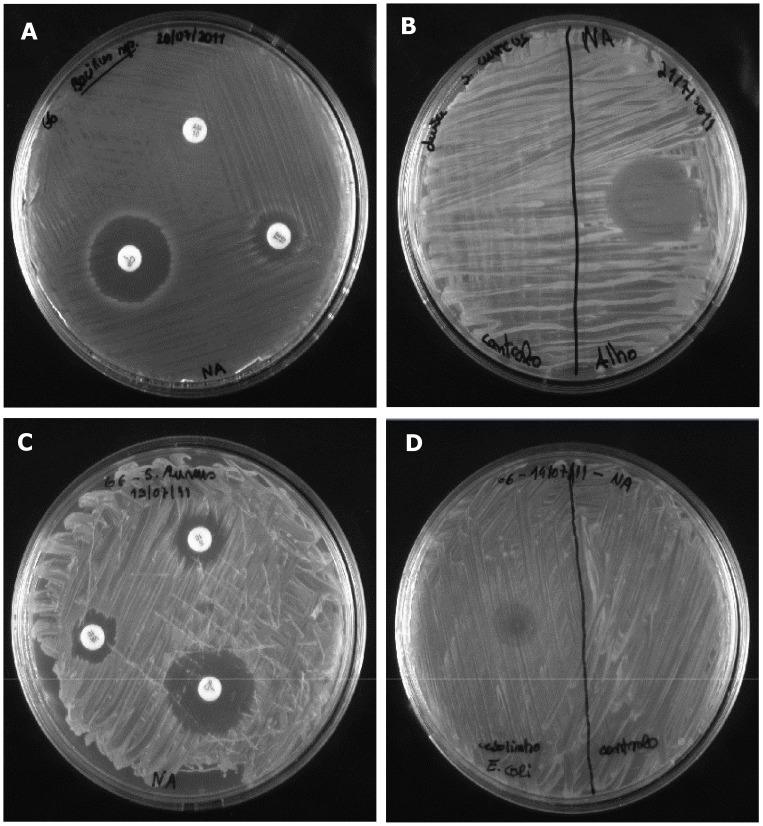
Positive results evidencing the procedural competencies of the participants. A – antibiograms obtained with commercial antibiotics – the even growth of the bacterial culture indicates an efficient inoculation; the clear inhibitory halos surrounding two antibiotic disks indicate that these were placed carefully onto the plates. B – Although the bacterial culture is not as evenly distributed as in ‘A’, its density allows the visualization of a halo in the point where a drop of garlic aqueous extract was applied (left side); the fact that the inhibitory halo is centered in relation to the half of the plate in which it was applied suggests the cautiousness of the participant who prepared the plate. Plates C and D were prepared by the same group of participants in consecutive days. The results evidence an improvement of their streaking technique. In C, an excess of inoculum appears to have been irregularly and incompletely distributed. The scratches in the medium (from the left to the middle of the plate) are suggestive of excessive pressure while streaking. In contrast, in D the inoculum is much more evenly distributed.

Most of the participants mentioned that they were unfamiliar with the laboratory procedures carried out and frequently asked questions about the surrounding laboratory equipment, wondering if it was “similar to the equipment available in research labs”. From the start, all of them were very careful in handling the materials and performing every procedure. Nevertheless, in addition to an evident enhancement in their self-confidence, their procedural competencies improved considerably along the week, as illustrated in Fig. 2. This was also observed for their engagement in the dry-lab activities. Although most of them had never experienced working with bioinformatics tools, they had no problems in following the protocols and discussing the issues raised.

#### Engagement and interaction

In spite of the expectable side talk, the participants were actively engaged in every task that they were asked to perform at a seemingly steady level throughout the week. They were eager to share their experiences as antibiotic consumers with their colleagues, and were particularly interested in how antibiotics are produced and act. They repeatedly mentioned that they were “going to share this information with relatives and friends”, because people “need to be alerted about this”.

The participants were quite excited about testing natural antibiotic compounds, and having the chance to get acquainted with new techniques and equipment.

Most of them were not acquainted with their colleagues, but by the second day all of them were working harmoniously. Everyone was called out by their names and cooperated with one another. The environment was light and easy-going, although there was some healthy competition between groups.

### Participants’ Activity Reports

While varying in structure and degree of detail, the participants’ reports included a description of the activities conducted and a summary of the main learning outcomes. No major errors or misconceptions were detected, although there was some confusion regarding the distinctiveness between horizontal and vertical gene transfer.

There were specific references to the fact that “resistance [genes] can be transmitted between bacteria”, and that “it is important to be careful about how antibiotics are used”. Some participants mentioned the social interaction that took place, stating that “the environment helped [them] to feel at ease” while engaging in the activities.

### Participants’ Feedback on the Project

The participants reported that they enjoyed participating in the project (*p*<0.001) and that it contributed to enhance their curiosity about the subjects addressed (*p*<0.001). They though that the contents were interesting (*p*<0.001), well-structured (*p*<0.001), and adequately articulated with the techniques (*p*<0.001). They believed that the project fostered the improvement of their understanding (*p*<0.001) and capacity to critically reflect about the issues discussed (*p*<0.001). Although considering that some effort was required for the successful completion of the activities (*p* = 0.002), they did not perceive the tasks proposed as too difficult (*p* = 0.69). Table S4 summarizes the participants’ mean responses to the feedback questionnaire.

The opportunity to practice new techniques and procedures (*n* = 25), to learn more about antibiotics and bacteria (*n* = 23), and the good environment and interaction that took place (*n* = 6), were highlighted as the most positive features of the project. In contrast, thirteen participants reported that the quality of the project could be improved by further increasing its practical component.

## Discussion

The project *Microbiology recipes: antibiotics à la carte* was a weeklong hands-on program designed to enhance the participants’ understanding about bacteria, antibiotics and antibiotic resistance. In line with this main goal, the data gathered reveals that this project provided the participants with a more elaborate picture of antibiotic resistance, by retooling them with more accurate conceptions about the interaction between bacteria and antibiotics, increasing their awareness about the perils of antibiotic resistance, and enabling them to bring into the equation a range of personal mitigating actions.

The quality of the participants’ responses was indicative of increased reasoning competencies (Table S2). Whereas high school students have been shown to have some difficulties in explaining biologic phenomena involving complex cause-effect relationships [46], the participants were able to mobilize notions that were not conveyed in the beginning of the project. They were able to cross-link elements that suggest a more thorough appraisal of individual actions to address some of the questions raised (as illustrated in Table S3). This was a very interesting outcome, considering that the main goal of the project was not to induce rote learning, but to empower the participants with concepts and scientifically sustained lines of reasoning to inform their decisions. That is why most questions allowed for responses within both the lower and higher levels of cognition defined by Bloom [41], [42]. Even if some only evoked knowledge and understanding (Q1, Q2, Q3, Q5 and Q8, Table S1), others also demanded analysis, synthesis and evaluation, among other competencies (Q4, Q6, Q7, Q9, Q10 and Q11, Table S1). As discussed in Lord & Baviskar [41], these emerged from the participants’ responses integrated in a continuum.

Previous studies have documented an array of misconceptions about bacteria, namely the confusion between these and other microorganisms, and the overestimation of their harmful effects [22], [26], [27], [47], [48]. At the beginning of the project, many participants already defined bacteria as unicellular, prokaryotic microorganisms, which is not surprising, given that they had just finished the 10^th^ and 11^th^ grades, the instructional levels in which the Portuguese biology curriculum comprises contents related with cell structure, uni−/multicellularity [28], taxonomy and classification [29]. What is interesting to find is that there was an increase in the quality of the definitions of bacteria provided by most of them at the end of the project. This is particularly important, considering that misconceptions about this notion can lead to misinformed behaviors [7], [25], [34]. For instance, it has been argued that the use of antibiotics for flu treatment can be a consequence of bacteria and viruses being misperceived as identical microorganisms [6], [7], [34], which is aggravated by a well-described tendency for physicians to prescribe antibiotics as a prophylactic approach to avoid latent and concomitant bacterial infections [49], [50]. When enquired about the adequacy of using antibiotics for flu treatment, the participants’ opinion shifted from the belief that these drugs are a suitable option, to the perception that this would be an inadequate line of treatment.

Contrasting with the reported tendency for bacteria to be associated with illness [22], [47], the participants acknowledged from the start that microorganisms can be both beneficial and harmful for humans. Nevertheless, following their participation in the project, they enhanced their repertoire of examples of bacteria that can have either effect. Moreover, whereas in the pre-test many of them believed that antibiotics only target pathogenic microorganisms, they ended up acknowledging that these drugs collaterally hinder part of the human microbiota. Taking into account the generalized lack of awareness towards the susceptibility of these vital communities to antibiotics [26], this is a relevant finding, as it may alert about the need to control antibiotic use.

Besides encouraging the use of antibiotics only when strictly necessary, the importance of following the physicians’ advice must be stressed, especially in what concerns finishing a full course of treatment [25], [26]. Having a general picture of the dynamics of bacterial growth can be insightful in this regard. Taking this into account, the bacterial growth curve was extensively discussed with the participants, leading to a substantial improvement of their description of the bacterial growth cycle. Most importantly, they understood that the knowledge about these aspects is necessary for adequate antibiotic use.

The participants’ conceptualization of antibiotics was also improved. Whilst many of them initially neglected the fact that these compounds only act on bacteria, most of them stated clearly in their post-tests that bacteria are the only target for antibiotic drugs. They also realized that each antibiotic has a more or less broad activity spectrum, and that their specificity is not limited to a particular bacterial species. Beyond providing the participants with a notion of what antibiotics are and how they act, the project was designed to evidence difficulties in counteracting and mitigating antibiotic resistance. A deeper evaluation of these difficulties can be obtained by appraising the complexity of the antibiotic production process, especially regarding the time involved, which barely allows keeping in pace with the rapid rate at which resistance spreads [51], [52], [53], [54] **.** These aspects were emphasized throughout the project**,** which, based on the data gathered, was shown to enhance the participants’ consciousness about the steps and difficulties in the development of new antibiotics.

Public misperceptions about antibiotics and bacteria extend to antibiotic resistance, which is often regarded as a feature of the host and not of the bacterium [25], [26]. To some extent contrasting with these reports, most of the participants were reasonably aware that antibiotic resistance refers to bacteria and not humans. But by the end of the project all of them acknowledged this aspect. Moreover, they called attention to the existence of resistance-related genes that can be transferred between bacteria. However, their success in answering correctly to question Q11 (“*Do you agree with the statement: The progeny of antibiotic resistant bacteria is also resistant? Justify your answer*”) was limited, since the distinction between horizontal and vertical gene transfer was somewhat misapprehended. Drawing on the weight of evidence pointing towards the major role of horizontal gene transfer in the dissemination of antibiotic resistance [53], [55], the project stressed the idea that antibiotic resistance genes can be interchanged between phylogenetically unrelated bacteria subjected to the selective pressure introduced by the same antibiotic [56]. The fact that this notion was not consistently manifested in the participants’ responses, suggests that some fine-tune adaptations of the instructional design and/or of the measurement instruments are required in future editions of the project. The participants were able to link antibiotic resistance with the improper use of antibiotics, which is a promising outcome, even if this improved understanding may not necessarily translate into adequate behaviors in this scope [34], [35].

The participants became more aware of measures to contain antibiotic resistance. Besides recognizing that the misuse and overuse of antibiotics has increased the number and diversity of resistant bacteria, they stressed the shortening of effective antibiotics and the difficulties in developing new ones. This awareness expectably fosters the recognition that judicious antibiotic use is fundamental [5], [26], [51]. Consistently, there were also noticeable changes in the participants’ beliefs about personal actions to address resistance. Interestingly, they went from identifying the production of new antibiotics as the only solution, to summarizing a series of individual behaviors that they though must be stimulated, as for instance avoiding self-medication, respecting the physician’s instructions, and reducing use. This outcome suggests that the project fostered the participants’ sense of self-responsibility, an essential condition for the success of any initiative aimed at promoting rational antibiotic use [26].

Concerning the factors underlying the effectiveness of this project, it is important to consider the influence of two major elements: the project’s instructional design and the environment in which it was implemented. This project has a marked hands-on character sustained by a meaningful and up-to-date body of scientific data and theory. In its design, particular attention was given to the balance and alignment of both components in each of the activities proposed. In fact, this aspect was highlighted by the participants, who expressed their satisfaction about the way in which practice and theory were integrated. Well contextualized practical activities are known to foster the ability to connect observable and conceptual dimensions [16], [19], [20]. Given that the project addresses biological processes that occur at microscopic and molecular levels, a possible explanation for the improvement of the participants’ conceptual understanding rests on the scaffolding provided by the visual outcomes and the diversified set of procedures involved in the activities.

Amongst its educational benefits, practical work is also expected to promote learning indirectly, by enhancing students’ interest [18], [57], [58], [59]. Accordingly, this project was intended to stimulate the participants’ short-term engagement with the contents and procedures, aiming to prompt their learning. Besides the observational field notes collected, the participants’ feedback reinforces the role of the practical tasks in engaging them in the activities. They mentioned that they valued the opportunity to practice their procedural skills, which is particularly important considering that most students do not get the chance to carry out practical work at their own schools [57], [60]. Furthermore, given that they consistently emphasized the interest and appeal of the activities, it is possible that this factor contributed to the improvement of their knowledge and understanding. It must be kept in mind that this interest was most likely situational [17], [61], deriving from the environment surrounding the participants. The summer school setting in which the project was implemented constitutes a key situational element that must be accounted for. UJr’s educational goals are placed within an informal, friendly, and relaxing environment of engagement and interaction, allowing it to be regarded as an educational leisure context. [32]. Studies have shown that these contexts harbor privileged opportunities for social interaction between the students, which may have a beneficial impact on their experience and learning [18], [19], [32]. Interestingly, many participants viewed the project precisely as an opportunity to interact with their peers, monitors and researchers, and associated this aspect with its success.

Finally, this study raises several questions that are worth pursuing in future research.

Based on the observations conducted, extending the study to a broader universe, focusing on diverse age groups, instructional levels and curricular backgrounds, should facilitate general conclusions. To avoid sampling biases, it would be particularly useful to implement the project in a formal classroom context. This was a case study with a small sample of high school science students who personally decided to enroll in the project. Therefore, although statistically significant results were obtained, these do not exclude the chance that these students might already nurture a personal interest about this topic, which might have made them more prone to engage in the activities. Nevertheless, this did not manifest in their baseline knowledge, which was not particularly robust. In turn, it raises the question of whether the magnitude of the improvements in the participants’ understanding would be identical if their baseline knowledge was sounder. It is also important to take into account that the project was implemented in a summer school setting, which, as mentioned above, can have impacted positively on the participants’ interest and learning. Implementing the project in formal settings would not only permit addressing this issue, but it would also grant the chance to embed the activities and contents in the students’ science curriculum, in articulation with the other school subjects. This should enable to distinguish the effects of traditional instruction practices from the outcomes of the activities. Having this in mind, the project’s activities can be easily adapted and implemented in schools. Moreover, besides being contextualized in the Portuguese biology curricula [28], [29], the concepts addressed are covered in science curricula from other countries, including the National Science Education Standards [62].

Another aspect to consider relates to the subjective nature of the qualitative data gathered through the open-ended questions in the pre- and post-tests. Although these were required to identify subtle variations in the quality of the participants’ reasoning [63], their interpretation is open to subjectivity, regardless of the thoroughness of the content analysis performed. The notions provided by the participants in response to the tests used in this study can be applied to the development of closed questions to be used in future quantitative studies.

Future research could also look into the influence of the project on the participants’ antibiotic use behaviors. This study was set up following a pre−/post-test design, in which the post-test was applied immediately after the completion of the project. Therefore, the findings must not be extrapolated beyond its framework. Especially considering that the study was not devised to evaluate long-term retention of information, and the improvements identified in the participants’ knowledge and understanding do not necessarily imply positive modifications on their antibiotic use behaviors. In fact, the association between knowledge about antibiotics and antibiotic use is not utterly demonstrated, given the contrasting evidence conveyed in different studies [7], [34], [35]. The assessment of this specific dimension can be achieved through a long-term longitudinal study to track the impact of these activities by the time these teenagers reach adulthood and engage in decision-making concerning antibiotic use.

Overall, this and other projects alike represent a contribution to enhance the consciousness about judicious antibiotic use amongst future generations. In addition, the insights made available in this study extend beyond the topic specificity of the project, by evidencing the educational benefits of incorporating hands-on activities in science education programs.

## Supporting Information

Figure S1 **Two examples of protocols provided to the participants.** These protocols illustrate some procedures conducted in the scope of a (A) wet lab activity and a (B) dry lab activity. See Figure 1 for the full list of activities implemented.
(TIF)

Figure S2 **Microbiology recipes: antibiotics à la carte project plan.** The activities were implemented as suggested in this figure, although their schedule and planning can be adapted and altered.
(TIF)

Table S1 **Pre−/post-test scoring rubrics used to assess the participants’ understanding and beliefs about bacteria, antibiotics and antibiotic resistance.**
(DOCX)

Table S2 **Pre−/post-test variations in the quality of the participants’ responses.**
(DOCX)

Table S3 **Notions and claims provided in the pre-test and in the post-test.**
(DOCX)

Table S4 **Participants’ feedback on the activity.**
(DOCX)
