# Supplementary figures and images for: Increasing Awareness about Antibiotic Use and Resistance: A Hands-On Project for High School Students
Source: PLoS One. 2012 Sep 12;7(9):e44699. doi: 10.1371/journal.pone.0044699 (PMC3440366; doi:10.1371/journal.pone.0044699)

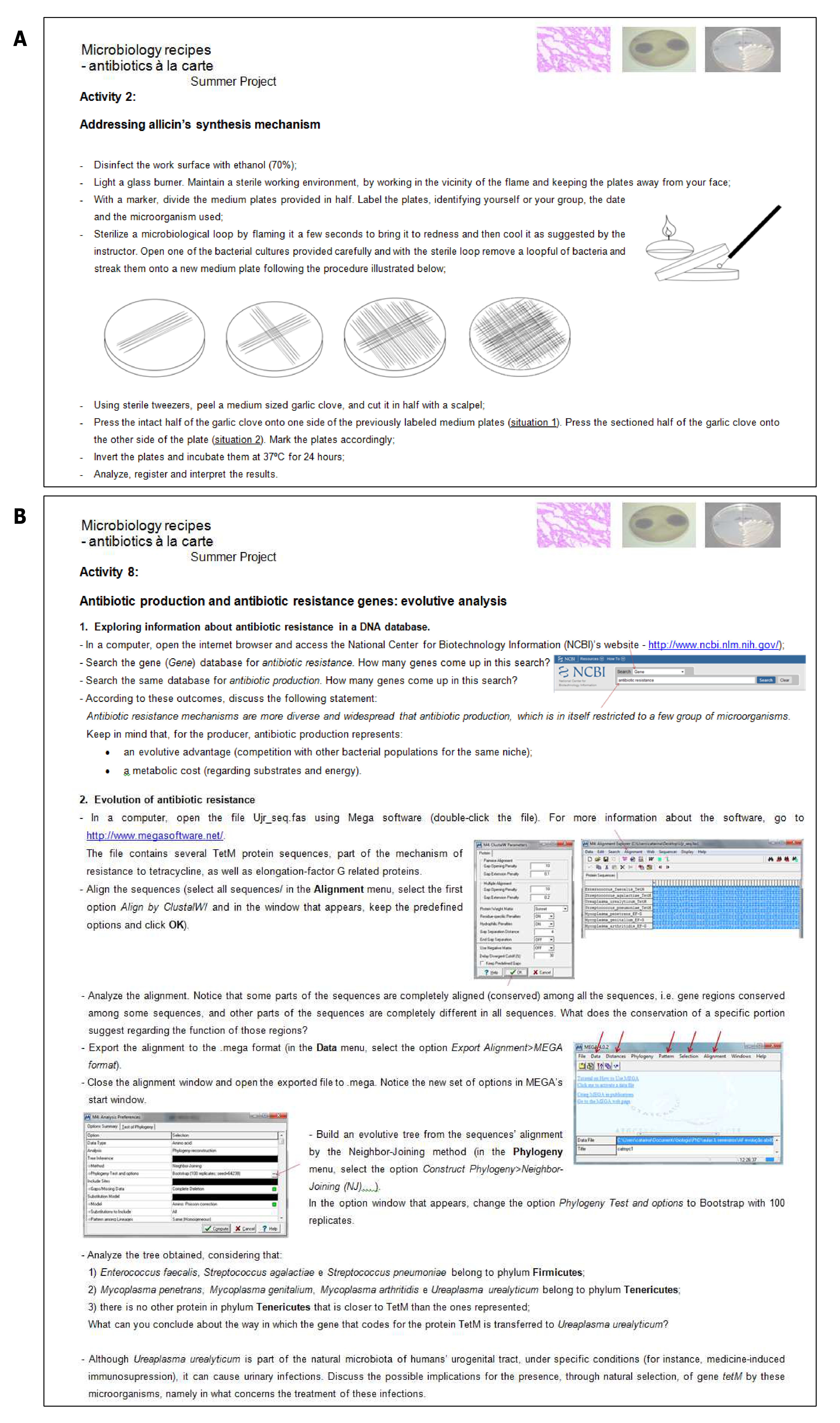

Supplement: Figure S1 — Two examples of protocols provided to the participants. These protocols illustrate some procedures conducted in the scope of a (A) wet lab activity and a (B) dry lab activity. See Figure 1 for the full list of activities implemented. (TIF) [file pone.0044699.s001.tif]

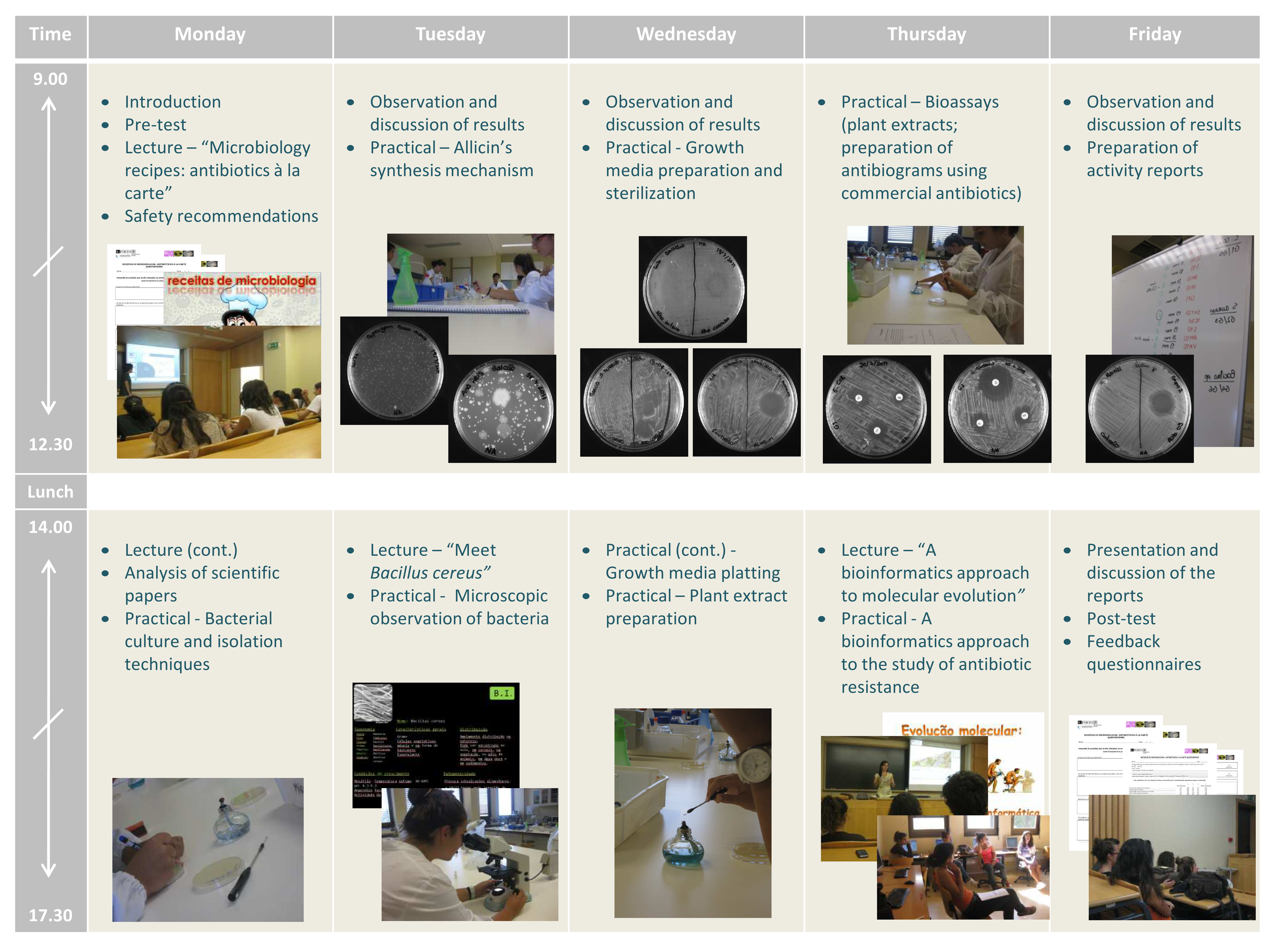

Supplement: Figure S2 — Microbiology recipes: antibiotics à la carte project plan. The activities were implemented as suggested in this figure, although their schedule and planning can be adapted and altered. (TIF) [file pone.0044699.s002.tif]
