# Supplementary material for: Increasing Awareness about Antibiotic Use and Resistance: A Hands-On Project for High School Students
Source: PLoS One. 2012 Sep 12;7(9):e44699. doi: 10.1371/journal.pone.0044699 (PMC3440366; doi:10.1371/journal.pone.0044699)
Supplement: Table S1 — Pre−/post-test scoring rubrics used to assess the participants’ understanding and beliefs about bacteria, antibiotics and antibiotic resistance. (DOCX) [file pone.0044699.s003.docx]

**Table S1. Pre-/post-test scoring rubrics used to assess the participants’ understanding and beliefs about bacteria, antibiotics and antibiotic resistance.**

| **Q1. How do you define bacteria?** | | | | | |
| --- | --- | --- | --- | --- | --- |
| Bloom’s Level | Criteria | | | Points | Qualitative classifier |
| Knowledge (LO)  Comprehension (LO) | - No answer - Only incorrect notions - More incorrect than correct notions (one or two notions) | | | 0 | Inadequate/ failing effort |
|  | - Only one correct notion - Two correct notions and one incorrect - More incorrect than correct notions (three or more notions) | | | 1 | Impaired/ minimum effort |
|  | - Same amount of correct and incorrect notions (three or more notions) - Two correct notions and none incorrect | | | 2 | Needs much improvement/ low effort |
|  | - More correct than incorrect notions (three or more notions) | | | 3 | Needs improvement/ effort |
|  | - Three or more correct notions and none incorrect (only notions related with bacteria morphology and phylogeny, e.g. microorganism, prokaryote) | | | 4 | Adequate/ good effort |
|  | - Three or more correct notions and none incorrect (including notions related with bacteria physiology and activity, e.g. pathogenic, antibiotic resistant) | | | 5 | High achievement/ high effort |
| **Q2. Are bacteria beneficial or harmful for humans? Give some illustrative examples.** | | | | | |
| Bloom’s Level | Criteria | | | Points | Qualitative classifier |
| Knowledge (LO)  Comprehension (LO) | - No answer - None example or justification or both incorrect - One or two correct examples and one incorrect - Bacteria as exclusively beneficial/ harmful, one correct and one incorrect example | | | 0 | Inadequate/ failing effort |
|  | - Bacteria as beneficial and/or harmful, and one correct example - Bacteria as exclusively beneficial/ harmful, and more correct than incorrect examples (two or more examples) | | | 1 | Impaired/ minimum effort |
|  | - Bacteria as exclusively beneficial/ harmful, and the same amount of correct and incorrect examples (two or more examples) - Bacteria as beneficial and harmful, and one correct example or more incorrect than correct examples (two or more examples) | | | 2 | Needs much improvement/ low effort |
|  | - Bacteria as exclusively beneficial/ harmful, and more correct than incorrect examples (two or more examples) - Bacteria as beneficial and harmful, and the same amount of correct and incorrect examples (two or more examples) | | | 3 | Needs improvement/ effort |
|  | - Bacteria as exclusively beneficial/ harmful, two or more correct examples and none incorrect - Bacteria as beneficial and harmful, and more correct than incorrect examples (two or more examples) | | | 4 | Adequate/ good effort |
|  | - Bacteria as beneficial and harmful, three to six correct examples, and none incorrect | | | 5 | High achievement/ high effort |
|  | - Bacteria as beneficial and harmful, more than six correct examples, and none incorrect | | | 6 | Outstanding achievement/ very high effort |
| **Q3. Describe the main phases in bacteria’s growth cycle.** | | | | | |
| Bloom’s Level | | Criteria | | Points | Qualitative classifier |
| Knowledge (LO)  Comprehension (LO) | | - No answer - Only incorrect notions - Identification of one or two phases, but no description | | 0 | Inadequate/ failing effort |
|  | | - Identification and description (or only description) of one or two phases - One or more correct notions | | 1 | Impaired/ minimum effort |
|  | | - Identification and description (or only description) of one or two phases, and one or more correct notions - Identification of three phases, and one or more correct notions - Identification and description of three phases, but one or more incorrect notions - Identification of four phases, but one or more incorrect notions | | 2 | Needs much improvement/ low effort |
|  | | - Identification and description of three phases, or identification of four phases | | 3 | Needs improvement/ effort |
|  | | - Identification and description of three phases, or identification of the four phases, and two or more correct notions | | 4 | Adequate/ good effort |
|  | | - Identification and description (or only description) of the four phases, two or more correct notions, but some incorrect notions as well | | 5 | High achievement/ high effort |
|  | | - Identification and description of the four phases and two or more correct notions | | 6 | Outstanding achievement/ very high effort |
| **Q4. Do you think that bacterial infectious diseases are currently under control? Justify your answer.** | | | | | |
| Bloom’s Level | | | Criteria | Points | Qualitative classifier |
| Knowledge (LO)  Comprehension (LO)  Application (HO)  Synthesis (HO)  Evaluation (HO) | | | - No answer - No justification or only inadequate justifications | 0 | Inadequate/ failing effort |
|  | | | - Valid and coherent explanation with one adequate claim | 1 | Needs much improvement/ low effort |
|  | | | - Valid and coherent explanation with two or three adequate claims | 2 | Adequate/ good effort |
|  | | | - Valid and coherent explanation with more than three adequate claims | 3 | High achievement/ high effort |
| **Q5. How do you define antibiotics?** | | | | | |
| Bloom’s Level | | | Criteria | Points | Qualitative classifier |
| Knowledge (LO)  Comprehension (LO) | | | - No answer - Only incorrect notions - More incorrect than correct notions (one or two notions) | 0 | Inadequate/ failing effort |
|  | | | - Only one correct notion - Two correct notions and one incorrect - More incorrect than correct notions (three or more notions) | 1 | Impaired/ minimum effort |
|  | | | - Same amount of correct and incorrect notions (three or more notions) - Two correct notions and none incorrect | 2 | Needs much improvement/ low effort |
|  | | | - More correct than incorrect notions (three or more notions) | 3 | Needs improvement/ effort |
|  | | | - Three to five correct notions and none incorrect | 4 | Adequate/ good effort |
|  | | | - More than five correct notions and none incorrect | 5 | High achievement/ high effort |
| **Q6. How do you explain the selectivity of antibiotics for microorganisms?** | | | | | |
| Bloom’s Level | | | Criteria | Points | Qualitative classifier |
| Knowledge (LO)  Comprehension (LO)  Application (HO)  Evaluation (HO) | | | - No answer - Only incorrect notions - One or two correct notions but more than two incorrect notions | 0 | Inadequate/ failing effort |
|  |  |  | - Only one correct notion - Two correct and two incorrect notions | 1 | Impaired/ minimum effort |
|  | | | - Two correct notions and one incorrect | 2 | Needs much improvement/ low effort |
|  | | | - Suitable response with two correct notions (difference between prokaryotic and eukaryotic cells, specificity and concentration of antibiotic compounds) | 3 | Adequate/ good effort |
| **Q7. Imagine that you have the flu, you are feverish and aching. In this situation, do you think that antibiotic prescription would be a suitable solution? Justify your answer.** | | | | | |
| Bloom’s Level | | | Criteria | Points | Qualitative classifier |
| Knowledge (LO)  Comprehension (LO)  Application (HO)  Synthesis (HO)  Evaluation (HO) | | | - No answer - Yes or No without justification | 0 | Inadequate/ failing effort |
|  |  |  | - Yes, but with adequate justification (e.g. control of concomitant and secondary bacterial infections) | 1 | Impaired/ minimum effort |
|  | | | - No, with adequate justification including one correct notion - No, with adequate justification including more than one correct notions, but also one or more incorrect notions | 2 | Needs much improvement/ low effort |
|  | | | - No, with adequate justification including only correct notions | 3 | Adequate/ good effort |
| **Q8. Describe how an antibiotic is produced.** | | | | | |
| Bloom’s Level | | | Criteria | Points | Qualitative classifier |
| Knowledge (LO)  Comprehension (LO) | | | - No answer - Only incorrect notions | 0 | Inadequate/ failing effort |
|  | | | - One correct notion - Two or three correct notions, but one or two incorrect | 1 | Impaired/ minimum effort |
|  | | | - Two or three correct notions - Four to six correct notions, but two or more incorrect | 2 | Needs improvement/ effort |
|  | | | - Four to six correct notions - More than six correct notions, but two or more incorrect | 3 | Adequate/ good effort |
|  | | | - More than six correct notions, and none incorrect | 4 | High achievement/ high effort |
|  | | | - More than six correct notions, following a reasonable line of reasoning | 5 | Outstanding achievement/ very high effort |
| **Q9. How do you define antibiotic resistance?** | | | | | |
| Bloom’s Level | | | Criteria | Points | Qualitative classifier |
| Knowledge (LO)  Comprehension (LO)  Application (HO) | | | - No answer - Only incorrect notions - More incorrect than correct notions (one or two notions) | 0 | Inadequate/ failing effort |
|  | | | - Only one correct notion - Two correct notions and one incorrect - More incorrect than correct notions (three or more notions) | 1 | Impaired/ minimum effort |
|  | | | - Same amount of correct and incorrect notions (three or more notions) - Two correct notions and none incorrect | 2 | Needs much improvement/ low effort |
|  | | | - More correct than incorrect notions (three or more notions) | 3 | Needs improvement/ effort |
|  | | | - Three or more correct notions and none incorrect (only notions related with the definition of resistance, e.g. a feature of the bacterium, allows bacteria to survive in the presence of the antibiotic compound) | 4 | Adequate/ good effort |
|  | | | - Three or more correct notions and none incorrect (including notions related with mechanisms of resistance and transfer of resistance, e.g. alternative metabolic pathways, horizontal and vertical gene transfer) | 5 | High achievement/ high effort |
| **Q10. List measures that can be used to avoid or reduce antibiotic resistance.** | | | | | |
| Bloom’s Level | | | Criteria | Points | Qualitative classifier |
| Knowledge (LO)  Application (HO) | | | - No answer - Only incorrect notions | 0 | Inadequate/ failing effort |
|  | | | - Only one correct notion - Two or three correct notions, but one or two incorrect | 1 | Impaired/ minimum effort |
|  | | | - Two correct notions and none incorrect - Three or four correct notions, but two or more incorrect | 2 | Needs improvement/ effort |
|  | | | - Three or four correct notions and none incorrect - More than five correct notions, but two or more incorrect | 3 | Adequate/ good effort |
|  | | | - More than five correct notions and none incorrect | 4 | High achievement/ high effort |
| **Q11. Do you agree with the statement: *The progeny of antibiotic resistant bacteria is also resistant*. Justify your answer.** | | | | | |
| Bloom’s Level | | | Criteria | Points | Qualitative classifier |
| Knowledge (LO)  Application (HO)  Application (HO)  Analysis (HO)  Evaluation (HO) | | | - No answer - Only incorrect notions | 0 | Inadequate/ failing effort |
|  | | | - Only one correct notion - Two or three correct notions, but one or two incorrect | 1 | Impaired/ minimum effort |
|  | | | - Two correct notions and none incorrect - Three or four correct notions, but two or more incorrect | 2 | Needs improvement/ effort |
|  | | | - Three or four correct notions (that may not be the most adequate to fully address the question) and none incorrect - More than four correct notions, but two or more incorrect | 3 | Adequate/ good effort |
|  | | | - More than four correct notions (the most adequate to address the question, e. g. There are antibiotic resistance-related genes in the genomic DNA and in the plasmids, while plasmids may not be transmitted to the daughter-cells, genes in the genomic DNA are), but two or more incorrect notions | 4 | High achievement/ high effort |
|  | | | - More than four correct notions (the most adequate to address the question) and none incorrect | 5 | Outstanding achievement/ very high effort |

“Don’t know” answers were considered as “No answer” and attributed no points. See Supporting file Table S3 for examples of notions conveyed in the pre- and post-test.
