# Supplementary material for: Increasing Awareness about Antibiotic Use and Resistance: A Hands-On Project for High School Students
Source: PLoS One. 2012 Sep 12;7(9):e44699. doi: 10.1371/journal.pone.0044699 (PMC3440366; doi:10.1371/journal.pone.0044699)
Supplement: Table S2 — Pre−/post-test variations in the quality of the participants’ responses. (DOCX) [file pone.0044699.s004.docx]

**Table S2. Pre-/post-test variations in the quality of the participants’ responses.**

| **Q1. How do you define bacteria?** | | | | | | | | | | |
| --- | --- | --- | --- | --- | --- | --- | --- | --- | --- | --- |
|  |  |  | | Paired Samples *t*-test | | | | Effect size | | |
|  |  | *M ± SD* | | *t*(41) | | *p* | | *d* | | |
| Scoring rubric score | Pre-test | 1.95 *±* 1.32 | | 4.86 | | 0.00 | | 0.90 | | |
|  | Post-test | 3.19 *±* 1.44 | |  | |  | |  | | |
| Number of correct notions/ response | Pre-test | 2.10 *±* 1.36 | | 3.55 | | 0.00 | | 0.65 | | |
|  | Post-test | 3.00 *±* 1.40 | |  | |  | |  | | |
| Number of incorrect notions/ response | Pre-test | 0.36 *±* 0.79 | | -2.80 | | 0.01 | | 0.53 | | |
|  | Post-test | 0.05 *±* 0.22 | |  | |  | |  | | |
|  | | | | | | | | | | |
| **Q2. Are bacteria beneficial or harmful for humans? Give some illustrative examples.** | | | | | | | | | | |
|  |  |  | | Paired Samples *t*-test | | | | Effect size | | |
|  |  | *M ± SD* | | *t*(41) | | *p* | | *d* | | |
| Scoring rubric score | Pre-test | 2.31 ± 1.73 | | 5.24 | | 0.00 | | 0.99 | | |
|  | Post-test | 3.86 ± 1.39 | |  | |  | |  | | |
| Number of correct examples of beneficial bacteria/ response | Pre-test | 0.69 ± 0.60 | | 3.12 | | 0.00 | | 0.62 | | |
|  | Post-test | 1.21 ± 1.03 | |  | |  | |  | | |
| Number of correct examples of harmful bacteria/ responses | Pre-test | 0.71 ± 0.46 | | 3.47 | | 0.00 | | 0.72 | | |
|  | Post-test | 1.19 ± 0.83 | |  | |  | |  | | |
|  | | | | | | | | | | |
| **Q3. Describe the main phases in bacteria’s growth cycle.** | | | | | | | | | | |
|  |  |  | | Paired Samples *t*-test | | | | Effect size | | |
|  |  | *M ± SD* | | *t*(41) | | *p* | | *d* | | |
| Scoring rubric score | Pre-test | 0.19 ± 0.40 | | 11.34 | | 0.00 | | 2.62 | | |
|  | Post-test | 3.83 ± 1.92 | |  | |  | |  | | |
| Number of correct notions/ response | Pre-test | 0.33 ± 0.65 | | 13.97 | | 0.00 | | 3.12 | | |
|  | Post-test | 7.17 ± 3.03 | |  | |  | |  | | |
|  | | | | | | | | | | |
| **Q4. Do you think that bacterial infectious diseases are currently under control? Justify your answer.** | | | | | | | | | | |
|  |  |  | | Paired Samples *t*-test | | | | Effect size | | |
|  |  | *M ± SD* | | *t*(41) | | *t*(41) | | *d* | | |
| Scoring rubric score | Pre-test | 0.61 ± 0.77 | | 5.49 | | 0.00 | | 1.18 | | |
|  | Post-test | 1.54 ± 0.81 | |  | |  | |  | | |
| Number of valid claims to support the response | Pre-test | 0.52 ± 0.67 | | 6.95 | | 0.00 | | 1.43 | | |
|  | Post-test | 1.86 ± 1.14 | |  | |  | |  | | |
| Number of invalid claims to support the response | Pre-test | 0.62 ± 0.58 | | -5.87 | | 0.00 | | 1.06 | | |
|  | Post-test | 0.12 ± 0.33 | |  | |  | |  | | |
|  | | | | | | | | | | |
| **Q5. How do you define antibiotics?** | | | | | | | | | | |
|  |  |  | | Paired Samples *t*-test | | | | Effect size | | |
|  |  | *M ± SD* | | *t*(41) | | *p* | | *d* | | |
| Scoring rubric score | Pre-test | 0.88 ± 1.21 | | 7.18 | | 0.00 | | 1.39 | | |
|  | Post-test | 2.93 ± 1.70 | |  | |  | |  | | |
| Number of correct notions/ response | Pre-test | 1.14 ± 0.85 | | 7.04 | | 0.00 | | 1.42 | | |
|  | Post-test | 3.48 ± 2.13 | |  | |  | |  | | |
| Number of incorrect notions/ response | Pre-test | 0.71 ± 0.71 | | -2.47 | | 0.02 | | 0.51 | | |
|  | Post-test | 0.38 ± 0.58 | |  | |  | |  | | |
|  | | | | | | | | | | |
| **Q6. How do you explain the selectivity of antibiotics for microorganisms?** | | | | | | | | | | |
|  |  |  | | | Paired Samples *t*-test | | | | Effect size | |
|  |  | *M ± SD* | | *t*(41) | | *p* | | *d* | | |
| Scoring rubric score | Pre-test | 0.17 ± 0.54 | | 2.05 | | 0.04 | | 0.39 | | |
|  | Post-test | 0.43 ± 0.77 | |  | |  | |  | | |
| Number of correct notions/ response | Pre-test | 0.14 ± 0.42 | | 2.71 | | 0.01 | | 0.49 | | |
|  | Post-test | 0.40 ± 0.63 | |  | |  | |  | | |
|  | | | | | | | | | | |
| **Q7. Imagine that you have the flu, you are feverish and aching. In this situation, do you think that antibiotic prescription would be a suitable solution? Justify your answer.** | | | | | | | | | | |
|  | | | | Paired Samples *t*-test | | | | Effect size | | |
|  |  | *M ± SD* | | *t*(41) | | *p* | | *d* | | |
| Scoring rubric score | Pre-test | 0.24 ± 0.76 | | 7.10 | | 0.00 | | 1.55 | | |
|  | Post-test | 1.81 ± 1.21 | |  | |  | |  | | |
| Number of correct notions/ response | Pre-test | 0.14 ± 0.47 | | 6.79 | | 0.00 | | 1.36 | | |
|  | Post-test | 1.10 ± 0.88 | |  | |  | |  | | |
| Number of incorrect notions/ response | Pre-test | 0.31 ± 0.47 | | -2.22 | | 0.03 | | 0.33 | | |
|  | Post-test | 0.17 ± 0.38 | |  | |  | |  | | |
|  | | | | | | | | | | |
| **Q8. Describe how an antibiotic is produced.** | | | | | | | | | | |
|  |  |  | | Paired Samples *t*-test | | | | Effect size | | |
|  |  | *M ± SD* | | *t*(41) | | *p* | | *d* | | |
| Scoring rubric score | Pre-test | 0.26 ± 0.70 | | 7.89 | | 0.00 | | 1.58 | | |
|  | Post-test | 1.95 ± 1.34 | |  | |  | |  | | |
| Number of correct notions/ response | Pre-test | 0.33 ± 0.93 | | 7.43 | | 0.00 | | 1.49 | | |
|  | Post-test | 2.95 ± 2.30 | |  | |  | |  | | |
|  | | | | | | | | | | |
| **Q9. How do you define antibiotic resistance?** | | | | | | | | | | |
|  |  |  | | Paired Samples *t*-test | | | | Effect size | | |
|  |  | *M ± SD* | | *t*(41) | | *p* | | *d* | | |
| Scoring rubric score | Pre-test | 2.71 ± 1.94 | | 4.02 | | 0.00 | | 0.76 | | |
|  | Post-test | 3.98 ± 1.37 | |  | |  | |  | | |
| Number of correct notions/ response | Pre-test | 2.33 ± 1.39 | | 3.62 | | 0.00 | | 0.77 | | |
|  | Post-test | 3.38 ± 1.34 | |  | |  | |  | | |
| Number of incorrect notions/ response | Pre-test | 0.19 ± 0.40 | | -3.11 | | 0.00 | | 0.67 | | |
|  | Post-test | 0.00 ± 0.00 | |  | |  | |  | | |
|  | | | | | | | | | | |
| **Q10. List measures that can be used to avoid or reduce antibiotic resistance.** | | | | | | | | | | |
|  |  |  | Paired Samples *t*-test | | | | Effect size | | |  |
|  |  | *M ± SD* | | *t*(41) | | *p* | | *d* | | |
| Scoring rubric score | Pre-test | 0.64 ± 0.73 | | 8.85 | | 0.00 | | 1.79 | | |
|  | Post-test | 2.21 ± 1.00 | |  | |  | |  | | |
| Number of correct notions/ response | Pre-test | 0.67 ± 0.72 | | 8.23 | | 0.00 | | 1.72 | | |
|  | Post-test | 2.45 ± 1.27 | |  | |  | |  | | |
|  | | | | | | | | | | |
| **Q11. Do you agree with the statement: *The progeny of antibiotic resistant bacteria is also resistant*. Justify your answer.** | | | | | | | | | | |
|  |  |  | | | Paired Samples *t*-test | | | | Effect size | |
|  |  | *M ± SD* | | *t*(41) | | *p* | | *d* | | |
| Scoring rubric score | Pre-test | 0.48 ± 0.74 | | 3.19 | | 0.00 | | 0.54 | | |
|  | Post-test | 1.00 ± 1.13 | |  | |  | |  | | |
| Number of correct notions/ response | Pre-test | 0.52 ± 0.74 | | 4.27 | | 0.00 | | 0.73 | | |
|  | Post-test | 1.21 ± 1.12 | |  | |  | |  | | |
| Number of incorrect notions/ response | Pre-test | 0.24 ± 0.43 | | 2.67 | | 0.01 | | 0.43 | | |
|  | Post-test | 0.45 ± 0.55 | |  | |  | |  | | |

*M* ± *SD* – Mean ± Standard Deviation. *d* – Cohen’s *d* measure of effect size. The scoring rubrics used to rate the participants’ responses can be found in Supporting file Table S1, and a list of notions conveyed in the pre- and post-test is available in Supporting file Table S3.
