# Supplementary material for: Increasing Awareness about Antibiotic Use and Resistance: A Hands-On Project for High School Students
Source: PLoS One. 2012 Sep 12;7(9):e44699. doi: 10.1371/journal.pone.0044699 (PMC3440366; doi:10.1371/journal.pone.0044699)
Supplement: Table S3 — Notions and claims provided in the pre-test and in the post-test. (DOCX) [file pone.0044699.s005.docx]

**Table S3. Notions and claims provided in the pre-test and in the post-test.**

| **Q1. How do you define bacteria?** | | | | |  |
| --- | --- | --- | --- | --- | --- |
|  | *n* | | McNemar test | |  |
|  | Pre-test | Post-test | *χ*^2^ | *p* |  |
| Correct notions |  |  |  |  |  |
| Unicellular | 21 | 31 | 5.06 | 0.02 |  |
| Beneficial or harmful | 6 | 16 | 5.79 | 0.01 |  |
| Monera kingdom | 6 | 15 | 5.82 | 0.01 |  |
| Incorrect notions |  |  |  |  |  |
| Multicellular | 6 | 0 | 4.17 | 0.03 |  |
|  |  |  |  |  |  |
| **Q2. Are bacteria beneficial or harmful for humans? Give some illustrative examples.** | | | | |  |
|  | *n* | | McNemar test | | |
|  | Pre-test | Post-test | *χ*^2^ | *p* | |
| Correct examples of beneficial bacteria |  |  |  |  | |
| Human symbiotic bacteria | 10 | 25 | 11.53 | 0.00 | |
| Correct examples of harmful bacteria |  |  |  |  | |
| *Bacillus cereus* | 0 | 13 | 11.08 | 0.00 | |
| Disease | 19 | 7 | 6.72 | 0.01 | |
|  |  |  |  |  | |
| **Q3. Describe the main phases in bacteria’s growth cycle.** | | | | |  |
|  | *n* | | McNemar test | | |
|  | Pre-test | Post-test | *χ*^2^ | *p* | |
| Growth phases |  |  |  |  | |
| Identification |  |  |  |  | |
| Lag | 0 | 27 | 25.04 | 0.00 | |
| Exponential | 0 | 32 | 30.03 | 0.00 | |
| Stationary | 0 | 30 | 28.03 | 0.00 | |
| Death | 0 | 31 | 29.03 | 0.00 | |
| Four phases | 0 | 29 | 27.03 | 0.00 | |
| Description |  |  |  |  | |
| Lag | 0 | 18 | 16.06 | 0.00 | |
| Exponential | 0 | 30 | 28.03 | 0.00 | |
| Stationary | 0 | 28 | 26.04 | 0.00 | |
| Death | 0 | 28 | 28.03 | 0.00 | |
| Four phases | 0 | 18 | 16.06 | 0.00 | |
| Correct notions |  |  |  |  | |
| Death | 4 | 29 | 21.33 | 0.00 | |
| Exponential growth | 0 | 23 | 21.04 | 0.00 | |
| Influence of the medium conditions | 1 | 13 | 8.64 | 0.00 | |
| Incorrect notions |  |  |  |  | |
| Confusions regarding the lag phase (e.g. "when the bacterium arrives at the medium") | 0 | 6 | 4.17 | 0.03 | |
|  |  |  |  |  | |
| **Q4. Do you think that bacterial infectious diseases are currently under control? Justify your answer.** | | | | |  |
|  | *n* | | McNemar test | | |
|  | Pre-test | Post-test | *χ*^2^ | *p* | |
| Students answering: |  |  |  |  | |
| Yes, bacterial infectious diseases are contained | 17 | 4 | 9.600 | 0.00 | |
| No, bacterial infectious diseases are not contained | 12 | 31 | 15.43 | 0.00 | |
| Valid claims |  |  |  |  | |
| Bacteria may be resistant to certain antibiotics | 5 | 33 | 26.04 | 0.00 | |
| Bacteria adapt | 2 | 14 | 8.64 | 0.00 | |
| The number of efficient antibiotics is limited and it is difficult to develop new ones | 1 | 11 | 8.10 | 0.00 | |
| Invalid claims |  |  |  |  | |
| There are sterilization mechanisms and information | 14 | 0 | 12.07 | 0.00 | |
|  |  |  |  |  | |
| **Q5. How do you define antibiotics?** | | | | |  |
|  | *n* | | McNemar test | | |
|  | Pre-test | Post-test | *χ*^2^ | *p* | |
| Correct notions |  |  |  |  | |
| Inhibits bacterial growth | 5 | 29 | 20.35 | 0.00 | |
| Kills bacteria | 6 | 33 | 25.04 | 0.00 | |
| Modes of action | 1 | 8 | 40.00 | 0.02 | |
| Produced by bacteria or fungus | 0 | 7 | 5.14 | 0.02 | |
| Incorrect notions |  |  |  |  | |
| Confusion about bacteriostatic (e.g. "inhibits several metabolic processes in bacteria") | 0 | 6 | 4.17 | 0.03 | |
|  |  |  |  |  | |
| **Q6. How do you explain the selectivity of antibiotics for microorganisms?** | | | | |  |
|  | *n* | | McNemar test | | |
|  | Pre-test | Post-test | *χ*^2^ | *p* | |
| Correct notions |  |  |  |  | |
| Specificity of action | 5 | 14 | 5.82 | 0.01 | |
| Incorrect notions |  |  |  |  | |
| Confusion between selectivity and spectrum of activity | 16 | 29 | 7.68 | 0.00 | |
|  |  |  |  |  | |
| **Q7. Imagine that you have the flu, you are feverish and aching. In this situation, do you think that antibiotic prescription would be a suitable solution? Justify your answer.** | | | | |  |
|  | *n* | | McNemar test | | |
|  | Pre-test | Post-test | *χ*^2^ | *p* | |
| Students answering: |  |  |  |  | |
| Yes, antibiotics can be used for flu treatment | 17 | 8 | 3.77 | 0.05 | |
| No, antibiotics cannot be used for flu treatment | 14 | 32 | 11.12 | 0.00 | |
| I do not know if antibiotics can be used for flu treatment | 11 | 2 | 7.11 | 0.00 | |
| Correct notions |  |  |  |  | |
| The flu is caused by a virus | 1 | 19 | 11.45 | 0.00 | |
| Antibiotics act on bacteria | 1 | 17 | 14.06 | 0.00 | |
| The overuse of antibiotics can lead to increased resistance | 0 | 11 | 9.09 | 0.00 | |
|  |  |  |  |  | |
| **Q8. Describe how an antibiotic is produced.** | | | | |  |
|  | *n* | | McNemar test | | |
|  | Pre-test | Post-test | *χ*^2^ | *p* | |
| Correct notions |  |  |  |  | |
| Isolation/ extraction of the compound | 2 | 24 | 18.38 | 0.00 | |
| Microbiological tests | 6 | 22 | 14.06 | 0.00 | |
| Concentration of the compound | 0 | 7 | 5.14 | 0.02 | |
| Animal tests | 1 | 19 | 16.06 | 0.00 | |
| Purification | 0 | 8 | 6.13 | 0.01 | |
| Clinical trials | 1 | 22 | 19.05 | 0.00 | |
| Formulation and commercialization | 2 | 12 | 8.10 | 0.00 | |
| Obtained from bacteria | 1 | 20 | 17.05 | 0.00 | |
| Use of compounds produced in laboratory | 0 | 9 | 7.11 | 0.00 | |
|  |  |  |  |  | |
| **Q9. How do you define antibiotic resistance?** | | | | |  |
|  | *n* |  | McNemar test | | |
|  | Pre-test | Post-test | *χ*^2^ | *p* | |
| Correct notions |  |  |  |  | |
| Feature of the bacterium | 31 | 40 | 7.11 | 0.00 | |
| Bacteria can adapt | 8 | 20 | 6.05 | 0.01 | |
| Incorrect notions |  |  |  |  | |
| Feature of the host | 6 | 0 | 4.17 | 0.03 | |
|  |  |  |  |  | |
| **Q10. List measures that can be used to avoid or reduce antibiotic resistance.** | | | | |  |
|  | *n* | | McNemar test | | |
|  | Pre-test | Post-test | *χ*^2^ | *p* | |
| Correct notions |  |  |  |  | |
| Avoid the overuse of antibiotics | 10 | 28 | 11.12 | 0.00 | |
| Respect the full length of treatment | 3 | 28 | 23.04 | 0.00 | |
| Avoid self-medication | 2 | 17 | 11.53 | 0.00 | |
| Respect the physician's instructions | 2 | 16 | 12.07 | 0.00 | |
|  |  |  |  |  | |
| **Q11. Do you agree with the statement: *The progeny of antibiotic resistant bacteria is also resistant*. Justify your answer.** | | | | |  |
|  | *n* | | McNemar test | | |
|  | Pre-test | Post-test | *χ*^2^ | *p* | |
| Correct notions |  |  |  |  | |
| There are antibiotic resistance-associated genes | 8 | 16 | 4.08 | 0.04 | |
| Vertical gene transfer | 8 | 24 | 12.50 | 0.00 | |
| Incorrect notions |  |  |  |  | |
| Confusion between vertical and horizontal gene transfer | 0 | 6 | 4.17 | 0.03 | |

*n* – number of participants who mentioned the notion. “Don’t know” answers were considered as “No answer”. A full list of notions provided by the participants is available from the authors upon request.
