# Supplementary material for: Increasing Awareness about Antibiotic Use and Resistance: A Hands-On Project for High School Students
Source: PLoS One. 2012 Sep 12;7(9):e44699. doi: 10.1371/journal.pone.0044699 (PMC3440366; doi:10.1371/journal.pone.0044699)
Supplement: Table S4 — Participants’ feedback on the activity. (DOCX) [file pone.0044699.s006.docx]

**Table S4. Participants’ feedback on the activity.**

|  |  | One Sample *t*-test | | Effect size |
| --- | --- | --- | --- | --- |
|  | *M ± SD* | *t*(41) | *p* | *d* |
| Difficulty of the contents | 3.07*±*0.71 | 0.65 | 0.52 | 0.14 |
| Interest of the contents | 4.52*±*0.55 | 17.90 | 0.00 | 3.91 |
| Organization and structuring of the contents | 4.17*±*0.80 | 9.33 | 0.00 | 2.07 |
| Difficulty of the techniques | 3.05*±*0.76 | 0.40 | 0.69 | 0,09 |
| Articulation between content and techniques | 4.40*±*0.70 | 13.00 | 0.00 | 2,83 |
| Suitability of the materials used | 4.52*±*0.80 | 12.29 | 0.00 | 2,69 |
| Effort required | 3.40*±*0.80 | 3.29 | 0.00 | 0,71 |
| Contribution to understand the issues discussed | 4.54*±*0.60 | 16.52 | 0.00 | 3,63 |
| Contribution to reflect critically about the issues discussed | 4.31*±*0.60 | 14.40 | 0.00 | 3,09 |
| Contribution to enhance the curiosity about the issues discussed | 4.60*±*0.67 | 15.55 | 0.00 | 3,38 |
| Satisfaction about the project | 4.40*±*0.67 | 13.70 | 0.00 | 2,96 |
| Evaluation of the project | 4.38*±*0.62 | 14.37 | 0.00 | 3,15 |

*M* ± *SD* – Mean ± Standard Deviation. *t* - one sample *t*-test (test value=3) for a 95% confidence interval. *d* - Cohen's *d* measure of effect size. Mean scores rated on a five-point Likert-type scale: 1- Very low/Not at all to 5 - Very high/Completely.
